# Cancer of the rectum

**DOI:** 10.1054/bjoc.2001.1768

**Published:** 2001-05

**Authors:** Y Bécouarn, M P Blanc-Vincent, M Ducreux, P Lasser, J B Dubois, M Giovannini, P Rougier

**Affiliations:** Institut Bergonié, Bordeaux; Cancer Research Campaign, Paris; Institut Gustave Roussy, Villejuif; Centre Val d'Aurelle Paul-Lamarque, Montpellier; Institut Paoli-Calmettes, Marseille; Hôpital Ambroise Paré, Boulogne, France


					Primary carcinoma of the rectum accounts for approximately 40%
of all colorectal carcinomas registered in France. The standardized
incidence of rectal cancer is among the highest in the world at 17.2
per 100 000 males and 8.6 per 100 000 for women.
The anatomical situation of cancer of the rectum presents two
particular problems: locoregional recurrence and sphincter preser-
vation. Specialized, multidisciplinary patient management is
therefore essential.
This document was validated in December 1998. An update is
planned for the year 2001.
DIAGNOSIS
The diagnosis of a rectal cancer is made by histological examina-
tion of a fragment of tumour obtained by endoscopic biopsy (stan-
dard).
STAGING
Locoregional staging depends on three examinations that are
cheap and possible in an outpatient setting: digital examination,
rigid rectoscopy and endorectal ultrasonography (standard). The
reliability of these examinations depends on the experience of
the examiner. A pelvic CT scan (which has replaced intravenous
urography) should be considered in the case of extensive disease,
as should MRI for large, deeply situated tumours (options).
Locoregional staging of a primary cancer of the rectum must be
carried out by an experienced multidisciplinary team.
Distant spread is assessed by clinical examination, chest X-ray
(AP and lateral), abdominal ultrasound and colonoscopy (stan-
dard). A CT scan of the liver can be undertaken if the ultrasound is
not satisfactory (option). The measurement of carcino-embryonic
antigen (CAE) levels is an option.
PROGNOSTIC FACTORS
The standard prognostic factors that enable a multidisciplinary
therapeutic decision to be made are: the presence or absence of
distant metastases, the type of rectal surgery undertaken (curative
or palliative), the extent of infiltration of the bowel wall by
tumour, invasion of adjacent organs by direct extension and the
presence or absence of metastatic locoregional nodes.
Other prognostic factors can add to the therapeutic decision-
making process. These include clinical factors such as presenta-
tion with acute bowel obstruction or perforation and pathological
factors such as venous or lymphatic invasion, local neural involve-
ment, the number of nodes examined and the number of metastatic
nodes detected.
A minimum of eight nodes should be removed for histological
examination (level of evidence C). Biological and genetic markers
should only be measured if their prognostic value is being evalu-
ated prospectively.
CLASSIFICATION
The most commonly used tumour classifications are postoperative
and based on pathological findings, even though therapeutic deci-
sions must be made before surgery. Therefore, prior to treatment,
the TNM classification of the International Union Against Cancer
(UICC), which includes information from staging endorectal ultra-
sonography is recommended. Following primary surgery, the
UICC pTNM post-therapeutic classification system takes into
account all the histopathological information on which further
treatment can be based.
TREATMENT MODALITIES
Surgery
In order to obtain satisfactory tumour clearance, the safe margin
between the lower end of the tumour and the rectal stump must be
greater than or equal to 2 cm (standard). A minimum of 6-8 nodes
should be examined (standard). Surgery with sphincter preserva-
tion (anterior resection or colo-anal anastomosis) is possible for
upper third cancers and usually for cancers of the middle third of
the rectum (option).
A radical resection (abdomino-perineal (A-P) resection) is
usually required for tumours of the lower third of the rectum
(option). If an A-P resection is undertaken, epiplooplasty to fill the
perineal wound is recommended (level of evidence B). For
tumours of the lower third of the rectum, or the middle third but
palpable by digital examination, excision of the entire mesorectum
reduces the risk of locoregional recurrence (level of evidence C).
Patients with cancers of the lower third of the rectum should be
included in surgical protocols evaluating sphincter preservation.
There is currently no indication for extensive pelvic nodal clear-
ance with the aim of reducing the risk of tumour recurrence (level
of evidence B). Procedures conserving the erector nerves, to try
and improve a patient's quality of life, are not recommended, as an
adverse effect on local tumour control has not yet been excluded
(level of evidence B). In cases of colo-anal or colorectal anasto-
moses, the construction of a colonic pouch to replace the rectal
reservoir improves the functional outcome for patients (level of
evidence B).
After surgical resection, the pelvis should be packed. This
reduces the risk of major complications for patients receiving post-
operative irradiation by preventing the interposition of small
Cancer of the rectum
Y Becouarn1
, MP Blanc-Vincent1,2
, M Ducreux3
, P Lasser3
, JB Dubois4
, M Giovannini5
and P Rougier6
1
Institut Bergonie, Bordeaux; 2
FNCLCC, Paris; 3
Institut Gustave Roussy, Villejuif; 4
Centre Val d'Aurelle Paul-Lamarque, Montpellier; 5
Institut Paoli-Calmettes,
Marseille; 6
Hopital Ambroise Pare, Boulogne, France
69
British Journal of Cancer (2001) 84 (Supplement 2), 69-73
(c) 2001 FNCLCC
doi: 10.1054/ bjoc.2001.1768, available online at http://www.idealibrary.com on
intestine (level of evidence B). Laparoscopic surgery is only
recommended within prospective randomized studies.
Radiotherapy
Preoperative radiotherapy is indicated for T3 and T4 tumours of
the rectum (standard). Preoperative radiotherapy may be indicated
for T2 tumours, even though the risk of recurrence appears small
after complete surgical resection alone (option). A three- or four-
field technique is recommended (level of evidence B).
Radiotherapy is contra-indicated if pelvic irradiation has been
given previously (standard). Advanced age, psychiatric problems
and prior laparotomy are all relative contra-indications to preoper-
ative radiotherapy (option). The minimum recommended preoper-
ative dose is 45 Gy (level of evidence B).
Postoperative radiotherapy must be considered if surgical clear-
ance of tumour is incomplete in the absence of preoperative radio-
therapy, or if the tumour has been under-staged preoperatively
(standard). The minimum recommended postoperative dose is
50 Gy given as external-beam irradiation.
The place of intraoperative radiotherapy (with or without
external-beam radiotherapy) requires evaluation within random-
ized trials to assess both efficacy and toxicity and to define its role
in the treatment of rectal cancers.
Radiotherapy as sole treatment must only be undertaken in
patients with inoperable disease or surgical contra-indications. In
this case, radiochemotherapy is recommended.
The definitive choice of technique (external, intracavitary or
both) depends on the treatment facilities and the experience of the
radiotherapy service concerned.
Radiochemotherapy
Combination radiochemotherapy is recommended for the treat-
ment of locoregional disease in patients considered `inoperable'
because of medical reasons or if surgery is refused. Radiotherapy,
at a dose of  55 Gy, is combined with bolus chemotherapy (5-
fluorouracil (5-FU) + folinic acid, days 1-5 during weeks 1-5 of
radiotherapy), or with continuous infusion 5-FU (225 mg m-2
day-1
)
for the duration of radiotherapy. After 50 Gy, the operability of the
patient must be re-assessed.
Therapeutic trials are required to evaluate the place of neo-
adjuvant preoperative combination radiochemotherapy. These
must address such issues as the optimal efficacy/toxicity ratio
of the combination, the compliance with treatment and the
role of reportedly effective chemotherapy combinations when
given with radiotherapy. If a patient has had surgery and no preop-
erative irradiation, radiotherapy at a dose of 50 Gy, given in
conventional fractions combined with 5-FU given as a continuous
infusion, is recommended, especially if there are poor prognostic
factors.
Adjuvant chemotherapy
In the adjuvant situation, the indication for chemotherapy and/or
immunotherapy has not been standardized; no trial is specific for
cancers of the rectum. After resection of any node-positive tumour
chemotherapy with six courses of bolus 5-FU plus folinic acid
(high or low dose) days 1-5 can be considered (option), if the
patient cannot be included in a therapeutic trial evaluating routine
adjuvant chemotherapy.
Local treatment of early rectal cancers
Local treatment for small cancers of the rectum is not as well
defined as the more classical treatments. This form of treatment is
only appropriate for T1 or T2 tumours of  3 cm that are mobile,
node-negative on endorectal ultrasonography and histologically
well differentiated. Local treatment looks promising in some
studies (level of evidence C), but the results are often difficult to
interpret and must be confirmed. If possible, local transanal
surgery should be undertaken in order to obtain tissue for
histology. Close follow-up of these small cancers post-therapy is
essential in view of the risk of local recurrence and/or metastases
and because salvage treatment for recurrence is possible. Follow-
up should include clinical examination, rectoscopy, endorectal
ultrasonography and biopsy of any suspicious lesions. Local treat-
ment should only be considered for selected patients and delivered
by specialized teams (level of evidence C).
THERAPEUTIC STRATEGY
T1 N0 M0 tumours
Complete surgical resection with sphincter preservation by a
specialist surgeon is the standard treatment (Figures 1 and 2). For
T1 sub-peritoneal tumours, the total removal of the mesorectum
can be considered. Postoperative external radiotherapy, at appro-
priate dose, with or without extensive surgery, is indicated if the
histology of the excised section shows incomplete clearance
and/or the presence of metastatic nodes and/or invasion of the
perirectal fat (option).
T2 N0 M0 tumours
Primary, complete surgical resection with preservation of the
sphincter by a specialist surgeon should be undertaken if possible
(standard) (Figures 1 and 3). Preoperative external radiotherapy is
an option. If the local resection is histologically incomplete, addi-
tional treatment with radiotherapy with or without extensive
surgery can be undertaken (level of evidence C). For T2 sub-
peritoneal tumours, total removal of the mesorectum can be
considered.
T3 or T4 (or node-positive irrespective of T) M0
tumours
External radiotherapy followed by surgery is standard treatment
(Figures 1 and 4). Surgery preserving sphincter function should be
undertaken if possible, depending on the situation of the tumour in
relation to the sphincter, the volume of tumour and the anatomy of
the patient (narrow pelvis, obesity, etc) (standard). Removal of the
mesorectum can be considered for sub-peritoneal tumours
(option). After resection of a node-positive tumour, six courses of
bolus 5-FU/folinic acid days 1-5 can be considered (option).
Patients should be included in prospective randomized clinical
trials to determine the precise role and the optimal use of radio-
therapy and chemotherapy in this context, such as:
* preoperative chemotherapy and radiotherapy vs preoperative
radiotherapy alone
* adjuvant locoregional chemotherapy (intraportal or intraperi-
toneal) plus systemic chemotherapy vs systemic chemotherapy
alone
70 Y Becouarn et al
British Journal of Cancer (2001) 84 (Supplement 2), 69-73 (c) 2001 FNCLCC
* preoperative plus intraoperative radiotherapy vs preoperative
radiotherapy alone.
Synchronous, resectable (single or multiple)
asymptomatic hepatic or pulmonary metastases
Extended surgery can be undertaken. Concurrent rectal and hepatic
surgery is standard treatment if the hepatectomy involves less than
or equal to three segments (Figures 1 and 5). If this is not possible,
a partial hepatectomy can be carried out some time after the rectal
surgery, depending on the assessment of disease extent at that time
(option). Pulmonary surgery can be undertaken 3 months after the
rectal surgery, depending on the assessment of disease spread
(option). Additional pelvic radiotherapy according to local stage
can be considered if the resection has been complete. Primary
systemic chemotherapy, alone or in combination with radio-
therapy, can be considered before the rectal surgery (option).
Systemic chemotherapy in the interval between the rectal surgery
and the hepatic or pulmonary surgery can be considered (option).
Multiple synchronous symptomatic metastases
Palliative chemotherapy is the standard treatment (Figures 1 and 6).
This consists of biweekly 5-FU plus a continuous infusion of
folinic acid (the de Gramont protocol) with or without oxaliplatin
or irinotecan (options).
Specific local treatment is dependent on the clinical situation,
for example surgery for stoma formation, radiotherapy, laser
therapy, or chemotherapy plus radiotherapy (options). Systemic
chemotherapy can be combined with pelvic radiotherapy
(option). The 5-FU bolus/continuous infusion folinic acid proto-
cols (as above) are preferable for primary treatment (level of
evidence B).
Cancer of the rectum 71
British Journal of Cancer (2001) 84 (Supplement 2), 69-73
(c) 2001 FNCLCC
Cancer of the rectum-
metastatic at presentation?
yes
no
yes
no
yes
no
yes
no
yes
no
T1 N0 M0?
T2 N0 M0? See treatment of
T1 N0 M0
disease
(Figure 2)
See treatment of
T2 N0 M0
disease
(Figure 3)
See treatment of
multiple asymptomatic
non resectable
metastases
(Figure 7)
See treatment of
symptomatic
metastases
(Figure 6)
See treatment of
resectable
metastases
(Figure 5)
See treatment
of T3,T4 N0 M0
or
N+, all T, M0
disease
(Figure 4)
Symptomatic
metastases?
Resectable
metastases?
Figure 1 Therapeutic strategy for cancer of the rectum
T1 N0 M0 disease
Standard
surgical resection by an experienced surgeon
pathological examination of the operative sample
sphincter preservation if at all possible
Option
complete excision of the mesorectum for sub-
peritoneal tumours
Consider prognostic
factors
Resection microscopically incomplete
and/or metastatic nodes and/or
invasion of perirectal fat
no yes
Standards
there is no standard
Option
post-operative external beam radiotherapy +/-
extended surgery
Follow-up
Follow-up
Figure 2 Treatment of T1 N0 M0 disease
Synchronous, multiple, non-resectable, asymptomatic
metastases
There is no standard approach. Locoregional treatment (surgery
and/or radiotherapy) followed by palliative chemotherapy is
possible (option) (Figures 1 and 7). It is recommended that
patients be included in prospective randomized trials to determine
the precise role of the following therapies:
* hepatic intra-arterial chemotherapy for hepatic metastases
* systemic chemotherapy and pelvic radiotherapy
* palliative systemic chemotherapy.
FOLLOW-UP
Follow-up after potentially curative surgery for a rectal cancer has
the objective of detecting a recurrence or a second cancer as early
as possible.
A patient with a poor performance status who is not fit for
further surgery should undergo minimal follow-up. The standard
examinations for follow-up are: clinical examination, CXR, liver
ultrasound and colonoscopy. If the patient has had sphincter-
preservation surgery, rectoscopy and/or endorectal ultrasonog-
raphy can be done (option). If the interpretation of an ultrasound is
difficult, a CT scan of the pelvis and/or liver is an option. It is
recommended that patients be included in prospective randomized
studies to determine the best methods and frequency of follow-up
and in particular, to define the role of monitoring carcino-
embryonic antigen (CEA) levels.
72 Y Becouarn et al
British Journal of Cancer (2001) 84 (Supplement 2), 69-73 (c) 2001 FNCLCC
T2 N0 M0 disease
Standard
* surgical resection by an experienced surgeon
* pathological examination of the operative sample
* sphincter preservation if at all possible
Option
* preoperative external beam radiotherapy
* excision of the mesorectum for sub-peritoneal
tumours
Consider prognostic
factors
Resection microscopically incomplete
and/or metastatic nodes and/or
invasion of perirectal fat
no yes
Standard
there is no standard
Option
additional treatment with radiotherapy
+/- extended surgery
Follow-up
Follow-up
Figure 3 Treatment of T2 N0 M0 disease
T3,T4, N0 M0
or
N+ all T M0
disease
Standards
* extemal-beam radiotherapy followed by surgery
* sphincter conserving surgery if at all possible
Options
* complete excision of the mesorectum for sub-
peritoneal tumours
* for Dukes C disease, postoperative chemotherapy
(5-FU + folinic acid)
Follow-up
Figure 4 Treatment of non-metastatic T3 T4 N0 or N + all T disease
Cancer of the rectum metastatic at
presentation
Resectable metastases
Standard
simultaneous rectal and hepatic surgery (if the hepatectomy is to involve
3 or less segments)
Options
* hepatectomy 3 months after rectal surgery depending on the extent
of progression
* pulmonary surgery 3 months after rectal surgery depending on
the extent of progression
* neoadjuvant chemotherapy (inclusion of patients in therapeutic trials)
* external-beam radiotherapy has to be considered in case of complete
resection of the rectal tumour
Follow-up
Figure 5 Treatment of disease presenting with resectable metastases
Cancer of the rectum presenting with metastatic
disease
Symptomatic metastases
Standard
systemic palliative chemotherapy
Option
* 5-FU-FA or 5-FU bolus + methotrexate or raltitrexed or 5-FU-FA +
oxaliplatin or 5-FU-FA + irinotecan
* local treatment according to the clinical situation: stoma surgery,
radiotherapy, laser, chemotherapy + radiotherapy
* combination systemic chemotherapy and pelvic radiotherapy
Follow-up
Figure 6 Treatment of disease presenting with symptomatic metastases
CT scanning and MRI are not indicated as routine examinations
in the follow-up of rectal cancers. Liver function tests and markers
must not be measured as a routine. The observation of an elevated
CEA level must be confirmed by repeat testing after a minimal
interval of 1 month (standard). If a preoperative colonoscopy was
not done, or was incomplete, a postoperative colonoscopy should
be undertaken 6 months following treatment (standard). In all
cases, a colonoscopy should be done within the year of surgery and
repeated as indicated by the results (standard).
INTERNAL REVIEWERS
G Borel (Centre Paul Strauss, Strasbourg), T Conroy (Centre
Alexis Vautrin, Vandoeuvre-Les-Nancy), D de Raucourt (Centre
Francois Baclesse, Caen), JP Ghnassia (Centre Paul Strauss,
Strasbourg), JC Horiot (Centre Georges-Francois Leclerc, Dijon),
G Lorimier (Centre Paul Papin, Angers), P Maingon (Centre
Georges-Francois Leclerc, Dijon), C Marchal (Centre Alexis
Vautrin, Vandoeuvre-Les-Nancy), J Mihura (Centre Claudius
Regaud, Toulouse), G Monges (Institut Paoli Calmettes,
Marseille), TD Nguyen (Institut Jean Godinot, Reims), JC Ollier
(Centre Paul Strauss, Strasbourg), JP Pignon (Institut Gustave
Roussy, Villejuif), M Rivoire (Centre Regional Leon Berard,
Lyon), P Rouanet (Centre Val d'Aurelle, Montpellier),
C Schumacher (Centre Paul Strauss, Strasbourg), P Troufleau
(Centre Alexis Vautrin, Vandoeuvre-Les-Nancy) and P Wagner
(Centre Paul Strauss, Strasbourg).
EXTERNAL REVIEWERS
M Amouretti (Groupe Hospitalier Sud Haut l'Eveque, Pessac),
JF Bosset (CHRU Hopital Jean Minjoz, Besancon), O Bouche
(Hopital Robert Debre, Reims), J Faivre (CHRU Hopital du
Bocage, Dijon), M Gignoux (Hopital Cote de Nacre, Caen),
H. Johanet (Pontoise), F. Lazorthes (CHRU Hopital Purpan,
Toulouse), B Millat (CHU Hopital Saint-Eloi, Montpellier),
H Orfeuvre (Centre Hospitalier Fleyriat, Bourg-en-Bresse),
M Parneix (Hopital Saint-Andre, Jean Ababdie, Bordeaux),
E Pelissier (CHU Hopital Saint-Jacques, Besancon), A Quinton
(Groupe Hospitalier Saint-Andre Jean Ababdie, Bordeaux),
F Rothe-Thomas (Centre Hospitalier, Chambery), B Roullet
(Hopital Dupuytren, Limoges), JP Suchaud (Centre Hospitalier,
Roanne), G de Laroche (Clinique Mutualiste de la Digonniere,
Saint-Etienne), A Botton (Clinique Sainte-Marie, Pontoise),
E Calitchi (Boulogne), PL Etienne (Clinique Armoricaine, Saint-
Brieuc), Farcy-Jacquet (Clinique Armoricaine, Saint-Brieuc),
D Fric (Clinique du Mail, Grenoble), G Ganem (Centre Jean
Bernard, Le Mans), H Lauche (Clinique Clementville,
Montpellier), A Namias (Centre Gastro-enterologie, Pontoise) and
JL Toullec (Cabinet de Gastro-enterologie, La Rochelle).
Cancer of the rectum 73
British Journal of Cancer (2001) 84 (Supplement 2), 69-73
(c) 2001 FNCLCC
Cancer of the rectum presenting with metastatic disease
Multiple asymptomatic non resectable metastases
Standard
there is no standard
Option
locoregional treatment for the rectal tumour (surgery and/or
radiotherapy) then palliative chemotherapy for the metastases
Recommandation
inclusion of patients in therapeutic trials to evaluate:
* systemic chemotherapy and pelvic radiotherapy
* intra-arterial hepatic chemotherapy
* palliative chemotherapy
Follow-up
Figure 7 Treatment of disease presenting with asymptomatic non-
resectable metastases

				

